# The Role of Respiratory Viruses in Children with Ataxia-Telangiectasia

**DOI:** 10.3390/v13050867

**Published:** 2021-05-09

**Authors:** Ana Méndez-Echevarría, María Belén Caminoa, Teresa del Rosal, Inmaculada Casas, Francisco Pozo, Samuel Ignacio Pascual-Pascual, Mar García-Romero, Carmen Cámara, Cristina Calvo

**Affiliations:** 1 Department of Pediatric Infectious Diseases, La Paz University Hospital, 28046 Madrid, Spain; mabelencaminoa@gmail.com (M.B.C.); teredelrosal@yahoo.es (T.d.R.); ccalvorey@gmail.com (C.C.); 2 Translational Research Network in Pediatric Infectious Diseases, 28009 Madrid, Spain; icasas@isciii.es (I.C.); pacopozo@isciii.es (F.P.); 3 Respiratory Virus and Influenza Unit, CNM, Carlos III Health Institute, 28220 Madrid, Spain; 4 Neuropediatrics Department, La Paz University Hospital, 28046 Madrid, Spain; ipascual@salud.madrid.org (S.I.P.-P.); yambee@hotmail.com (M.G.-R.); 5 Immunology Department, La Paz University Hospital, 28046 Madrid, Spain; ccamarah@salud.madrid.org

**Keywords:** respiratory tract infections, viruses, rhinovirus, primary immunodeficiency diseases, ataxia-telangiectasia

## Abstract

Background: The impact of respiratory virus infection in patients diagnosed with ataxia-telangiectasia (A-T) has not been well studied. Methods: A prospective case control study was performed at a National Reference Unit for Primary Immunodeficiency in Spain (from November 2018 to July 2019), including patients younger than 20 years. Symptom questionnaires and nasopharyngeal swabs from multiple respiratory viruses’ polymerase chain reaction were collected monthly, and between visits in case of symptoms. Results: Twenty-two individuals were included (11 patients; 11 controls); 164 samples were obtained (81 patients; 84 controls). Patients presented respiratory symptoms more frequently compared with controls (26.5% vs. 3.5%; *p* < 0.01). Viral detection was observed in 23 (27.3%) episodes in patients and in 15 (17.8%) episodes in controls (*p* = 0.1). Rhinovirus was the most frequent virus in patients and controls (60% and 53.3%, respectively). Episodes with positive viral detection had associated symptoms in 54% of patients and 18% of controls (*p* = 0.07). However, patients with A-T presented a similar rate of symptoms during episodes with positive and negative viral detection (26% vs. 27%). The median points given for each questionnaire during symptomatic episodes with negative viral detection were 13/23 points, and during symptomatic positive detection, 7.5/23 points (*p* = 0.1). In the control group, all but two were asymptomatic during positive viral episodes (score: 2/23 and 3/23 points). Symptomatic episodes, with either positive or negative viral detection, were associated with lower IgA and higher IgM titers and higher CD8+ counts (*p* < 0.05), particularly when these episodes were moderate/severe. Conclusions: Patients with A-T more frequently present symptomatic viral infections than controls, especially those with lower IgA and higher IgM titers and higher CD8+ counts.

## 1. Introduction

Ataxia-telangiectasia (A-T) is an autosomal recessive, multiorgan disease characterized by progressive cerebellar ataxia, ocular telangiectasia, and variable defects in both humoral and cell-mediated immunity, with predisposition to infections and cancer [1,2,3].

Recurrent respiratory infections can result in progressive bronchiectasis and chronic lung disease [1,2,3,4]. Despite immunoglobulin replacement therapy (IRT) and/or antibiotic prophylaxis, recurrent infections are still one of the leading causes of morbidity and mortality in patients with A-T [1,3]. In addition, there is evidence of both inappropriate activations of the innate immune response and increased levels of pro-inflammatory cytokines in these patients, which could also influence the development of chronic lung disease [1].

The role of respiratory viruses in patients with A-T, which could favor bacterial co-infections, has hardly been studied. There are few data regarding the susceptibility to respiratory viruses in these children.

We report a prospective case control study that analyzes respiratory viral infections in pediatric patients with A-T, considering their relationship with their clinical symptoms and their immunological status.

## 2. Materials and Methods

We conducted a prospective case control single-center study for 9 months, from November 2018 to July 2019, in a National Reference Unit for Primary Immunodeficiency in Madrid, Spain.

The inclusion criteria were patients younger than 20 years of age, diagnosed with genetically confirmed A-T, who were being followed up in our center. For each patient, a healthy control was included, paired by sex and age. Respiratory samples (nasopharyngeal swabs) were obtained monthly from patients and controls. Every instance in which a respiratory sample was obtained was defined as an episode, regardless of the presence or absence of respiratory symptoms.

### 2.1. Clinical and Immunological Evaluation

Symptom questionnaires elaborated ad hoc were completed monthly [5], and in case of respiratory symptoms in patients and controls, recorded fever, increased respiratory secretions, cough, respiratory distress, sputum (increased production, change in its characteristics), absenteeism from school and/or work, need for steroids, bronchodilators, antibiotic therapy, and hospital admission. A score based on this questionnaire was established for each participant in each episode. Symptoms were quantified considering the scoring of questions from this questionnaire (Figure S1). Respiratory symptoms were classified as none/asymptomatic (0 points), mild (1–4 points), or moderate/severe (5 or more points). The score of the Scale for the Assessment and Rating of Ataxia (SARA) was recorded in all patients at inclusion.

A complete immunological study was performed for all cases at inclusion. In addition, immunoglobulin levels, total lymphocytes, and CD4+ and CD8+ counts were performed monthly in A-T cases. If patients/controls developed moderate/severe respiratory symptoms between visits, additional nasopharyngeal swabs were performed.

### 2.2. Microbiological Analysis

Nasopharyngeal swabs were sent for virological investigation to the Influenza and Respiratory Viruses Laboratory at the National Center for Microbiology, Madrid, Spain. Samples were processed within 24 h after collection. Upon reception, three aliquots were prepared and stored at −80 °C. Both the reception and the nasopharyngeal aspirate (NPA) sample processing areas were separate from those defined as working areas.

RNA and DNA from 200 μL NPA aliquots were extracted using the QIAamp Mini Elute Virus spin kit in an automated extractor (QIAcube, Qiagen, Valencia, Spain).

Respiratory virus detection was performed by four independent multiplex real-time polymerase chain reaction (RT-PCR) assays. The first assay detected influenza A, B, and C viruses; a second assay was used to detect parainfluenza viruses 1–4, human rhinovirus, and enteroviruses; and a third assay detected respiratory syncytial virus (RSV) types A and B, human metapneumovirus, human bocavirus, and adenovirus. These assays used the SuperScript™ III Platinum^®^ One-Step Quantitative RT-PCR System (Invitrogen). Human coronavirus was investigated using a generic RT-PCR, which was able to detect both alpha and beta coronaviruses. The primers and TaqMan probes that were employed had previously been reported by the study investigators [6]. The results from the nasopharyngeal swabs were reported to the investigative team at the end of the study.

### 2.3. Statistical Analysis

The values are expressed as percentages for discrete variables and as mean and standard deviation or median and interquartile range (IQR) for continuous variables. Categorical variables were compared using the chi-square and Fisher’s exact tests, and continuous variables with Student’s *t* test or nonparametric tests as appropriate. A 2-tailed value of *p* < 0.05 was considered statistically significant.

## 3. Results

During the study period, 11 patients with A-T and 11 healthy controls were included. Fifty-four percent (6/11) of the patients and controls (6/11) were male. The median age of the patients and healthy controls was 12.2 (IQR 9.8–15.7) and 13.2 (IQR 10–16.6) years, respectively (*p* = 0.9). Only one patient and one control were younger than 5 years.

Five patients were receiving IRT, and four were under cotrimoxazole prophylaxis due to low CD4 count. Eight patients were participating in a multicenter international clinical trial, which evaluates the efficacy of intra-erythrocyte dexamethasone versus placebo in patients with A-T (IEDAT-02-2015 Study) [7]. The immunological status at inclusion is described in Table 1, and the clinical characteristics of the included cases, as well as their treatments, are reported in Table 2.

Ultimately, 164 samples were obtained for respiratory virus analysis (81 episodes from patients and 84 from controls; 8 median samples per patient; IQR 6–9). Patients with A-T more frequently presented respiratory symptoms during the episodes (26.5%; 20/81) in comparison with controls (3.5%; 3/84; *p* < 0.01). Four additional nasopharyngeal swabs were performed in three patients due to moderate/severe respiratory symptoms between protocol visits. None of the controls required additional sampling due to moderate/severe respiratory symptoms. 

In patients with A-T, there were 23 (27.3%) episodes with a positive viral detection, whereas in healthy controls viruses were identified in 15 (17.8%) episodes (*p* = 0.1). Rhinovirus was the most frequently isolated virus in both cases (60%; 14/23 episodes) and controls (53.3%; 8/15 episodes). Complete microbiological results are shown in Table S1.

**Figure 1:**
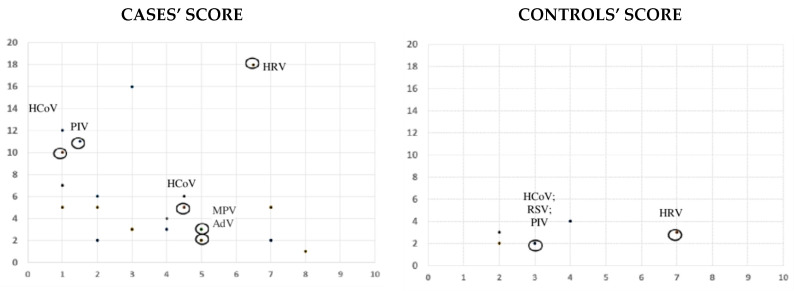
Case and control scores during symptomatic episodes; symptomatic episodes in which a virus was detected have been indicated with a circle. AdV: adenovirus; HCoV: human coronavirus; HRV: rhinovirus; MPV: metapneumovirus; PIV: parainfluenza virus; RSV: respiratory syncytial virus.

**Figure 2:**
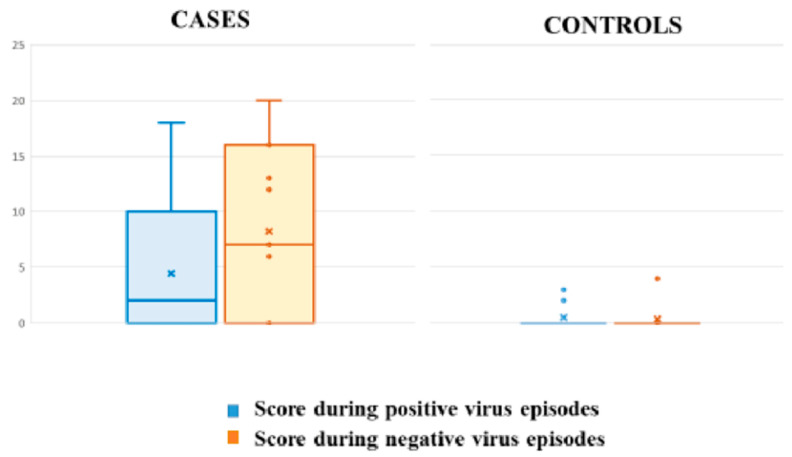
Score obtained from the symptoms questionnaire during positive and negative virus episodes in cases and controls. Box plots show minimum, maximum, median, and Q1 and Q3 questionnaire scores.

Eight patients with A-T (72%) and two controls (18%) presented respiratory symptoms during the study (*p* = 0.01), associated or not with respiratory virus isolation. Episodes with positive viral detection were associated with symptoms in 54% (6/11) of A-T cases and 18% (2/11) of controls (*p* = 0.07) (Figure 1). However, patients with A-T presented a high and similar rate of respiratory symptoms during episodes, both with positive and negative viral detection (26% (6/23) vs. 27% (17/61) (Figure 2). The median points given for each questionnaire during symptomatic episodes with negative viral detection were 13 points (IQR 7–16), and 7.5 points (IQR 2.7–18.7) during positive viral episodes (*p* = 0.1) (Figure 2). In the control group, all patients but two were asymptomatic during positive viral episodes. These two children presented mild symptoms, scoring 2 and 3 on the study questionnaire (Figure 1).

The median SARA (Scale for the Assessment and Rating of Ataxia) score in patients who presented with symptomatic viral episodes was 19.5 (IQR; 12.6–22.5), while that in the rest of the patients was 17.5 (IQR; 10–24.7) (*p* = 0.9). The median SARA score in patients who presented with symptoms during negative virus episodes was 18 (14-5-24), while that in other patients was 16 (IQR; 9.5–24.3) (*p* = 0.7).

Symptomatic episodes with positive viral detection were associated with lower IgA titers and higher lymphocyte counts (CD8+ and CD4+) (Table 3A). When these episodes were moderate/severe, lower IgA and higher IgM titers as well as higher CD8+ counts were observed (Table 3B). Symptomatic episodes with negative viral detection were also associated with lower IgA and higher IgM titers and higher lymphocyte counts (CD8+) (Table 3C).

Eight out of eleven patients reported recurrent respiratory infections before their inclusion in the study, with frequent use of antibiotics. Patients 4, 7, and 8 had no previously recurrent respiratory infections. During the study period, antibiotic prescription was observed in 9.5% (8/84) of the patients’ and 3.5% (3/84) of the controls’ episodes. In patients with A-T, antibiotics were prescribed in 13% (3/23) of episodes with positive viral detection and in 8% (5/61) with a negative one. Patients 10 and 11, who presented the highest score during non-viral episodes along the study period, had been admitted once due to suspected bacterial pneumonia before their inclusion, although microbiological studies were negative in both cases. None of the patients required hospital admission due to respiratory infection during the study period.

## 4. Discussion

Our results suggest that viral respiratory tract infections could play a role in children with A-T who present moderate respiratory symptoms, although these patients also present a high rate of symptomatic episodes without virus detection.Therefore, other mechanisms, including bacterial infections or intrinsic neurological mechanisms, rather than viruses, could also play a role in the respiratory manifestations of A-T patients. Lower IgA and higher IgM titers as well as higher CD8+ counts were significantly associated with symptomatic episodes, with or without viral detection.

Respiratory tract infections and chronic lung disease are an important cause of morbidity and mortality in A-T [1,2,3,4,8]. However, the etiology and pathogenesis of the chronic pulmonary disorders in these patients have not been well studied. Recurrent aspiration and poor pulmonary clearance due to abnormal muscle tone as well as an abnormal immune response could contribute to lung complications, due not only to an increased susceptibility to infections but also to a chronically heightened inflammatory state [1,2,8,9].

Bacteria are the main known causes of pneumonia in these patients, with *Staphylococcus aureus*, *Streptococcus pneumoniae,* and *Haemophilus influenzae* being the most frequently isolated microorganisms in younger patients with respiratory tract infections [2]. However, viral respiratory infections have barely been studied [3]. Case control prospective studies performed in patients with primary antibody deficiencies have reported worsening of symptom scores from the participant’s baseline, with the impaired lung function associated with respiratory viral infections. Rhinovirus was the virus most frequently detected, as we have also observed [5,10]. These studies included a heterogeneity of diseases, such as combined immunodeficiency or immune dysregulation diseases, with immunological profiles similar to A-T [5,10].

A-T is caused by mutations in the *ATM* gene that result in partial or complete loss of *ATM* activity [3]. Experimental studies have demonstrated that *ATM* activity plays an essential role in the innate immune response against respiratory viruses such as RSV or adenovirus [11,12]. This protein has a critical role in type I and III interferon (IFN) responses after RSV infection. Additionally, adenovirus and RSV replication could be enhanced in cells depleted of *ATM* [11,12]. Other authors have observed that *ATM*-null mice developed persistent peri-bronchial inflammation after recurrent influenza virus infections, revealing an absence of a proper memory response [9].

Bhatt et al. prospectively collected respiratory samples from 70 children with A-T for microbiological surveillance during scheduled non-urgent clinic visits, detecting one or more respiratory viruses in 31.8% of the samples, with rhinovirus being the most frequent [13]. In addition, Schroeder et al. observed lymphoid interstitial infiltrate in the lung biopsy of patients with A-T, similar to those found in patients with viral pneumonitis [2]. As we have reported, respiratory infections in patients with A-T are more severe and frequent than the ones observed in healthy individuals [3]. Although severe acute infections are uncommon in these patients, prolonged cough after viral infections is frequently observed [3].

Previous respiratory viral infection could also precipitate secondary bacterial infections, facilitating overgrowth of bacteria such as *H. influenzae* or *S. pneumoniae* [14,15]. In fact, *S. pneumoniae* is more frequently detected in the upper airway microbiome of patients with A-T than in healthy individuals [8]. Respiratory viruses could also have significant implications in the pathophysiology of respiratory complications in A-T [9,11,12,13] given that they are associated with substantial morbidity among other patients with primary immunodeficiency [5,6,7,8,9,10], although this has hardly been analyzed.

In our study, patients with symptomatic infections (with or without virus isolation) presented with lower IgA titers, higher IgM titers, and a higher CD8+ count. Other authors have also observed lower levels of IgA in patients with antibody deficiencies and respiratory virus infections compared with patients without virus detection [16]. Up to 10% of the patients with A-T have decreased IgA with normal to increased IgM levels, designated as a hyper-IgM (HIGM) phenotype [17]. This phenotype has shown a strongly reduced life expectancy in patients with A-T [18,19]. In these subgroups, respiratory failure is a common cause of death [3,19], with almost all patients developing recurrent upper and lower respiratory infections and chronic lung disease early in life [18,19]. Lymphoproliferative disorders are also more common among patients with A-T with an HIGM profile, reflecting immune dysregulation [17]. Some authors have reported higher rates of viral skin infections in them than in other patients with A-T [20]; however, there are no data on viral respiratory infections.

In addition to high IgM titers, the patients of our cohort with symptomatic respiratory infections presented higher CD8+ counts. Krauthammer et al. have reported a higher CD8+ count in 15 patients with HIGM compared with 31 non-HIGM cases, although this difference did not reach statistical significance (*p* = 0.09) [20]. Other studies did not observe this difference, although most of the published series included few patients [17,18,20]. The increase of CD8+ T cells in patients with viral respiratory infections could be explained by their role in the host defense against intracellular pathogens. However, we have also observed higher CD8+ counts in children with respiratory symptoms and negative virus results.

The presence of unrepaired DNA in *ATM*-deficient cells activates pathways causing the induction of type 1 IFNs, which lead to the inflammatory phenotype associated with A-T [1]. Other authors have hypothesized that *ATM*-deficient innate immune cells present an extended life due to altered DNA damage response, and could contribute to chronic inflammation, leading to the development of lung disease [9]. In addition, pro-inflammatory cytokines, such as interleukin-8 (IL-8), have been observed in patients with A-T, which could potentially cause tissue damage [8,21] and lead to the recruitment of a subset of CD8 T cells [22]. Some authors have suggested that respiratory viruses could be one of the triggers [3] that favor inflammation and lung damage in patients with A-T with interstitial lung disease [3]. Our findings are consistent with this inflammatory phenotype described in some patients [8,21].

Surprisingly, we also observed significantly elevated IgG titers in patients with symptomatic negative virus episodes. Given that many patients were under IRT, we are not able to interpret this finding, although it could also be caused by a pro-inflammatory state.

Several clinical trials suggest that steroids have a beneficial effect on neurological disability in A-T, with no significant effect on virus reactivation, using low-dose oral betamethasone or intra-erythrocyte infusion of dexamethasone [23,24]. In vitro dexamethasone has been shown to induce *ATM* restoration, which overcomes most of the mutations thus far described in the *ATM* gene [24]. However, its anti-inflammatory effects could contribute to patient improvement and are under investigation. The patients of our cohort who were included in the clinical trial “Intra-erythrocyte Infusion of Dexamethasone vs. Placebo” did not appear to present a poorer respiratory outcome, although our cohort was small and we did not know which patients were receiving placebo.

Cotrimoxazole was the prophylactic antibiotic prescribed in our cohort. No controlled trials have been performed in patients with A-T, and there is wide variation in the practice of prescribing prophylactic antibiotics in these patients [3]. Recent clinical trials performed in patients with common variable immunodeficiency have observed benefits of azithromycin prophylaxis, reducing respiratory exacerbations, hospital admissions, and antibiotic treatments [25]. This antibiotic has immunomodulatory and anti-inflammatory properties, reducing IL-8 concentrations and the chronic inflammation that leads to airway remodeling [25]. Pneumocystis infection is hardly ever diagnosed in patients with A-T [2,26], and progression of immunological abnormalities over time is unusual [3,13]. Other factors can also lead to long-term pulmonary complications, such as aspiration or recurrent viral infections [1,3,8,21]. In this context, azithromycin could be a better prophylactic option for most patients with A-T.

Our study has several limitations. Our cohort was small, given that A-T is a rare disease. We did not routinely perform other immunological evaluations, such as IgG subclass determination, which could offer additional relevant information, because patients with an HIGM profile usually have associated IgG2 deficiency [17]. Finally, we have not fully characterized those episodes with negative virus detection, given no bacterial cultures or respiratory function tests were performed. However, bacterial confirmation of lower respiratory infection in patients with A-T is difficult, because collecting high-quality samples is a challenge in this population. Other studies similar to ours were unable to obtain many scheduled samples because children with A-T would not cooperate or were unable to expectorate [13]. Difficulties in performing spirometry or cough peak flow in children with A-T are frequently observed in these patients, either because they are too young or a poor technique is employed [13].

Our results show how respiratory viruses, especially rhinovirus, can affect patients with A-T, especially those with lower IgA and higher IgM titers and higher CD8+ counts. However, other causes such as bacterial infection and/or neurological deficiencies could be the main cause of these respiratory manifestations. Further studies are needed to characterize the role of respiratory virus in the physiopathology of chronic lung disease in this group of patients.

## Figures and Tables

**Table 1:** Immunological studies of cases at the time of inclusion (*n* = 11).

Median Leukocyte Count	5890/µL (IQR; 3970–6540)
Median lymphocyte count	1140/µL (IQR; 910–1470)
Median T cells	
CD3+ % (count)	58% (670/µL; IQR 520–790)
CD4+ % (count)	29% (330/µL; IQR 240–440)
CD8+ % (count)	22% (250/µL; IQR 200–300)
Ratio of CD4+/CD8+	1.39 (IQR 0.93–1.66)
Median B cells CD19+ % (count)	7.8% (90/µL; IQR 50–130)
Median NK cells CD56+CD3- % (count)	35% (400/µL; IQR 270–740)
Median CD31+ (%)	4.1% (IQR 3.6–9%)
Median IgG *	986 mg/dL (IQR; 664–1300)
Median IgA	91 mg/dL (IQR; 0–127)
Median IgM	143 mg/dL (IQR; 101–235)
Reduced proliferative responses to mitogens (PHA, PWM) ^§^	4/10
Absence of tetanus antibody protective titers	5/11
Absence of pneumococcal antibody titers	8/11

* Five patients were under immunoglobulin replacement therapy; ^§^ PHA: phytohemagglutinin; PWM: pokeweed mitogen.

**Table 2:** The main clinical characteristics of included cases.

Patient	Age (Years)	Sex	SARA * Score	Clinical Trial IEDAT-02–2015	TMP-SMX Prophylaxis	IRT	% Symptomatic Episodes	% Viral Episodes	Total Score during Viral Episodes	Total Score during Non-viral Episodes
1	13	M	25.5	YES	NO	NO	25% (2/8)	37% (3/8)	2	16
2	10	M	11	YES	NO	NO	0% (0/8)	12% (1/8)	0	0
3	10	M	17.5	YES	YES	NO	33% (3/9)	11% (1/9)	0	13
4	16	F	9	YES	NO	NO	0% (0/5)	20% (1/5)	0	0
5	9	M	25.5	YES	NO	YES	0% (0/7)	42% (3/7)	0	0
6	12	F	21	YES	YES	YES	14% (1/7)	14% (1/7)	3	0
7	12	F	21.5	YES	NO	NO	20% (2/10)	50% (5/10)	5	6
8	3.5	M	7	NO	YES	NO	33% (3/9)	55% (5/9)	10	7
9	7	F	14.5	YES	YES	YES	44% (4/9)	22% (2/9)	18	12
10	15	M	24	NO	NO	YES	83% (5/6)	0% (0/6)	0	20
11	20	F	18	NO	NO	YES	66% (4/6)	16% (1/6)	11	16

F: female; IRT: immunoglobulin replacement therapy; M: male; SARA *: Scale for the Assessment and Rating of Ataxia; TMP-SMX: trimethoprim/sulfamethoxazole.

**Table 3:** (**A**) Comparison between symptomatic and asymptomatic patients during positive virus episodes; (**B**) between patients with and without moderate/severe symptoms during positive virus episodes; (**C**) between symptomatic and asymptomatic patients during negative virus episodes.

**3A**	**Symptomatic Patients during Virus Episodes (*n* = 5)**	**Asymptomatic Patients during Virus Episodes (*n* = 6)**	***p***
Cotrimoxazole prophylaxis	40% (2/6)	20% (1/5)	
Immunoglobulin replacement therapy	40% (2/6)	60% (3/5)	
Inclusion in clinical trial *IEDAT-02-2015*	50% (3/6)	80% (4/5)	
Median IgA levels during the study (mg/dL) (median; IQR)	33 (IQR 0–95)	109 (IQR 0–182)	*p* = 0.03
Median IgM levels during the study (mg/dL) (median; IQR)	145 (86–227)	151 (101–193)	*p* = 0.6
Median IgG levels during the study (mg/dL) (median; IQR)	956 (541–1250)	950 (887–1300)	*p* = 0.1
Blood lymphocyte count (cells/mm^3^) (median; IQR)	1365 (IQR; 1012–1825)	865 (IQR; 705–1125)	*p* = 0.0008
CD4+ /µL (cells/mm^3^) (median; IQR)	385 (282–482)	250 (190–320)	*p* < 0.001
CD8 + /µL (cells/mm^3^) (median; IQR)	260 (170-580)	200 (130-250)	*p* = 0.01
**3B**	**Patients with <5 survey points during virus episodes (*n* = 4)**	**Patients with ≥5 survey points during virus episodes (*n* = 7)**	***p***
Cotrimoxazole prophylaxis	50% (2/4)	28% (2/7)	
Immunoglobulin replacement therapy	50% (2/4)	42% (3/7)	
Inclusion in clinical trial *IEDAT-02-2015*	50% (2/4)	85% (6/7)	
Median IgA levels during the study (mg/dL) (median; IQR)	15 (IQR; 0–95)	91 (IQR; 32–133)	*p* = 0.008
Median IgM levels during the study (mg/dL) (median; IQR)	161 (IQR; 111–241)	125 (IQR; 86–189)	*p* = 0.04
Median IgG levels during the study (mg/dL) (median; IQR)	1120 (IQR; 664–1290)	939 (IQR; 538–1110)	*p* =0.3
Blood lymphocyte count (cells/mm^3^) (median; IQR)	1090 (IQR; 697–1472)	1090 (IQR; 842–1555)	*p* = 0.5
CD4+ /µL (cells/mm^3^) (median; IQR)	320 (230–437)	325 (220–432)	*p* = 0.9
CD8 + /µL (cells/mm^3^) (median; IQR)	460 (187–727)	200 (132–270)	*p* = 0.002
**3C**	**Symptomatic patients during NEGATIVE virus episodes (*n* = 7)**	**Asymptomatic patients during NEGATIVE virus episodes (*n* = 4)**	***p***
Cotrimoxazole prophylaxis	42% (3/7)	25% (1/4)	
Immunoglobulin replacement therapy	42% (3/7)	50% (2/4)	
Inclusion in clinical trial *IEDAT-02-2015*	57% (4/7)	100% (4/4)	
Median IgA levels during the study (mg/dL) (median; IQR)	15 (IQR; 0–95)	91 (IQR; 32–133)	*p* = 0.008
Median IgM levels during the study (mg/dL) (median; IQR)	166 (IQR; 130–230)	88 (IQR;65–104)	*p* < 0.001
Median IgG levels during the study (mg/dL) (median; IQR)	1085 (IQR; 864–1297)	561 (IQR; 463–953)	*p* = 0.003
Blood lymphocyte count (cells/mm^3^) (median; IQR)	1365 (825–1860)	1010 (842–1147)	*p* = 0.02
CD4+ /µL (cells/mm^3^) (median; IQR)	310 (250–380)	320 (197–440)	*p* = 0.60
CD8 + /µL (cells/mm^3^) (median; IQR)	280 (190–450)	180 (130–320)	*p* = 0.0003

## Data Availability

Not applicable.

## Supplementary Materials

The following are available online at https://www.mdpi.com/article/10.3390/v13050867/s1: Figure S1: Sympoms questionnaire. Table S1: Respiratory viruses identified during the study in patients and controls.
