# Supplementary material for: The Role of Respiratory Viruses in Children with Ataxia-Telangiectasia
Source: Viruses. 2021 May 9;13(5):867. doi: 10.3390/v13050867 (PMC8150715; doi:10.3390/v13050867)
Supplement: Supplementary file 1 [file viruses-13-00867-s001.zip › TABLE S1.pdf]

Supplementary file 2. *Respiratory viruses identified during the study in patients and controls*

PATIENTS	CONTROLS
8 Rhinovirus<br>2 HCoV-NL63<br>1 HCoV-OC43<br>1 Adenovirus<br>1 Influenza virus (H3N1)<br>1 Metapneumovirus<br>1 Coinfection: RSV; HCoV-229E; parainfluenza virus	13 Rhinovirus<br>2 HCoV-OC43<br>2 Adenovirus<br>2 Parainfluenza virus<br>2 Metapneumovirus<br>1 Enterovirus<br>1 Coinfection: Rhinovirus; HCoV-NL63

HCoV: human coronavirus; RSV: respiratory syncytial virus
